# Machine Learning for Predicting Human Drug-Induced Cardiotoxicity: A Scoping Review

**DOI:** 10.3390/toxics13121087

**Published:** 2025-12-17

**Authors:** Ja-Young Han, Min Jung Kim, Hyunwoo Kim, KeunOh Choi, Seongjin Ju, Myeong Gyu Kim

**Affiliations:** 1 Graduate School of Pharmaceutical Sciences, Ewha Womans University, Seoul 03760, Republic of Korea; httre555@naver.com (J.-Y.H.); minjung__kim@ewha.ac.kr (M.J.K.); 2 College of Pharmacy and Integrated Research Institute for Drug Development, Dongguk University-Seoul, Goyang 10326, Republic of Korea; hwkim8906@dongguk.edu; 3 Safety CoreLab Ltd., Seoul 06230, Republic of Korea; 4 College of Pharmacy, Ewha Womans University, Seoul 03760, Republic of Korea

**Keywords:** machine learning, cardiotoxicity, prediction model, scoping review

## Abstract

Background: Drug-induced cardiotoxicity poses a major challenge in drug development and clinical safety. Although machine learning (ML) methods have shown potential in predicting cardiotoxic risks, prior research has largely focused on specific mechanisms such as human Ether-à-go-go-Related Gene (hERG) inhibition. This scoping review systematically examined studies applying ML models to predict a broad range of drug-induced cardiotoxicity outcomes. Methods: A systematic search of PubMed, EMBASE, SCOPUS, and Web of Science identified studies developing ML models for cardiotoxicity prediction. Extracted data included sources, feature types, algorithms, and performance metrics, categorized by evaluation method (training, testing, cross-validation, or external validation). Results: Twenty-five studies met inclusion criteria, addressing outcomes such as arrhythmia, cardiac failure, heart block, hypertension, and myocardial infarction. Structured resources such as SIDER (Side Effect Resource) were the most common data sources, with features including molecular descriptors, fingerprints, and occasionally, target-based or transcriptomic data. Support vector machines (SVM) and random forest (RF) were the most common algorithms, showing robust predictive performance, with externally validated area under the receiver operating characteristic curve (AUC-ROC) values above 0.70 and accuracy exceeding 0.75 in several studies. Despite variability and limited external validation, ML approaches demonstrate substantial promise for predicting diverse cardiotoxic outcomes. Conclusions: This review underscores the importance of integrating heterogeneous data and rigorous validation for improving cardiotoxicity prediction.

## 1. Introduction

Cardiotoxicity is a major hurdle in drug development, often leading to discontinuation during preclinical and clinical stages, as well as post-market withdrawals [1,2]. While hepatotoxicity (18%) and immune-related reactions (17%) are the most commonly reported adverse drug reactions resulting in drug withdrawal, cardiotoxicity (14%) remains a critical concern due to its severe impact on heart function [3]. Defined as toxicity affecting the heart, cardiotoxicity encompasses both direct myocardial damage and structural impairment [4]. Its early detection is essential to ensure patient safety and to avoid the costly failure of drug development programs [5].

Machine learning (ML) algorithms are increasingly used in early drug development to predict compound toxicity by analyzing chemical structures and biological response data, thereby improving safety profiles and reducing late-stage failures [5,6] Among cardiotoxicity mechanisms, human Ether-à-go-go-Related Gene (hERG) potassium channel inhibition has been a major focus due to its association with QT prolongation and Torsades de Pointes (TdP) [7]. As such, predicting hERG blockade has become a standard component of preclinical safety assessment, and numerous in silico models, ranging from traditional Quantitative Structure-Activity Relationship (QSAR) to advanced ML algorithms, have been developed. Representative QSAR models for predicting hERG inhibition include freely available tools such as HergSpred (http://www.icdrug.com/ICDrug/T, accessed on 10 December 2025), AMED Cardiotoxicity (https://drugdesign.riken.jp/hERGdb/, accessed on 10 December 2025), ADMETlab (https://admetlab3.scbdd.com/, accessed on 10 December 2025), CardPred (http://bioanalysis.cau.ac.kr:7050/, accessed on 10 December 2025), Pred-hERG (https://predherg.labmol.com.br/, accessed on 10 December 2025), CardioDPI-Predictor (http://cardiodpi.sapredictor.cn/, accessed on 10 December 2025), and ProTox (https://tox.charite.de/protox3/, accessed on 10 December 2025), as well as various commercial software platforms that provide functionalities for hERG inhibition and cardiotoxicity prediction. Early ML models, such as Doddareddy’s support vector machine (SVM)-based approach using 2644 compounds, showed promising results [8]. In 2019, a study expanded this work using over 291,000 compounds, achieving high accuracy of 0.984 [9].

While hERG-centered models provide valuable insights, they capture only one aspect of drug-induced cardiotoxicity, and not all drugs that block hERG currents necessarily lead to arrhythmias [10]. Consequently, QSAR models targeting alternative mechanistic markers remain underdeveloped and require further refinement to fully capture the complexity of cardiac toxicity pathways [11,12]. Beyond arrhythmias, patients undergoing pharmacological treatment have also exhibited other cardiotoxic manifestations such as myocardial injury, ischemia, and hypertension [13]. Recent advances in computational toxicology have expanded the scope of in silico prediction to include a broader range of cardiotoxic effects, such as mitochondrial dysfunction, cardiomyocyte viability, and structural heart damage [10]. These models often integrate diverse data types, including transcriptomics, protein–protein interaction networks, and high-content screening assays, offering a more comprehensive evaluation of cardiac risk [14,15].

This scoping review aims to summarize ML models for predicting human drug-induced cardiotoxicity outcomes—including arrhythmia, cardiac failure, heart block, hypertension, and myocardial infarction—highlighting databases, input features, prediction targets, algorithms, and performance metrics to identify current trends and research gaps.

## 2. Methods

### 2.1. Study Selection

This scoping review was conducted in accordance with the PRISMA Extension for Scoping Reviews (PRISMA-ScR) (Table S1), and the review protocol was registered on the Open Science Framework (https://osf.io/9veru, accessed on 2 December 2025). Study selection followed a structured scoping review framework. A comprehensive literature search was conducted in PubMed, EMBASE, SCOPUS, and Web of Science on 22 November 2025, using predefined keywords related to cardiotoxicity and machine learning (Table 1). After removing duplicates, titles and abstracts were independently screened by two reviewers according to predefined inclusion and exclusion criteria. Full texts of potentially relevant studies were then assessed for eligibility. Disagreements were resolved through discussion or consultation with a third reviewer.

Studies were included if they met the following criteria: (1) the study utilized a dataset comprising at least 100 compounds; (2) the source of cardiotoxicity information was explicitly stated; (3) ML was applied to perform a binary classification of compounds as cardiotoxic or non-toxic; (4) the study addressed at least one human cardiotoxicity outcome, including arrhythmia, cardiac failure, heart block, hypertension, or myocardial infarction; and (5) the study reported quantitative performance metrics such as accuracy, sensitivity (recall), specificity, precision, F1 score, area under the receiver operating characteristic curve (AUC-ROC), or Matthews correlation coefficient (MCC). Eligible publication types included peer-reviewed journal articles, conference proceedings, and articles in press. Studies were excluded if they focused solely on hERG channel activity as the cardiotoxicity endpoint. In addition, review articles, editorial letters, notes, conference abstracts, and book chapters were excluded from this review.

### 2.2. Data Extraction and Synthesis

The extracted data included author names, publication year, database sources, input features, dataset size, algorithms used, evaluation metrics, and key findings. Information was collected from the text, tables, and figures of each study. To ensure consistency and accuracy, two reviewers independently performed data extraction, with discrepancies resolved through discussion or consultation with a third reviewer. When numerical results were not explicitly provided, values were obtained using digital extraction tools or estimated from graphical representations when possible. The findings from all included studies were synthesized descriptively to highlight key trends, methodological characteristics, and overall outcomes.

In this scoping review, reported model performance metrics were categorized based on the type of dataset used for evaluation: training dataset, testing dataset, cross-validation, and external validation. The training dataset refers to the subset of data used to fit the model parameters. The testing dataset is an internal hold-out subset drawn from the same source as the training data but not used during training. Cross-validation typically involves partitioning the training data into multiple folds to iteratively train and validate the model. External validation involves evaluating the trained model on entirely independent datasets collected from different sources, serving as the most stringent test of model generalizability. For studies that evaluated multiple models, only the performance metrics of the best-performing model were extracted and summarized in our review.

### 2.3. Study Quality and Risk-of-Bias Assessment

To contextualize reported model performance and mitigate the risk of over-interpreting results derived from heterogeneous or insufficiently documented datasets, we performed a structured study quality/risk-of-bias assessment tailored to ML-based classification studies of cardiac toxicity. The assessment focused on data credibility, validation rigor, and reproducibility. Specifically, we evaluated whether each study (1) clearly reported the data provenance; (2) described and appropriately handled class imbalance; (3) conducted evaluation on an independent external test set; (4) reported a confusion matrix or sufficient information to reconstruct key counts; (5) supported reproducibility by providing code; and (6) provided explainability analyses (e.g., feature importance or mechanistic interpretation) that linked model inputs to endpoints. Each domain was rated as Low, Moderate, or High risk using prespecified criteria.

Data provenance was rated as Low risk when the full source of the data was clearly reported, Moderate risk when only partial information was provided, and High risk when no such information was available. Handling of class imbalance was rated as Low risk when imbalance was explicitly acknowledged and appropriately addressed, and High risk when no handling or discussion was reported. The validation strategy was considered Low risk when evaluation was performed on an independent external test set, Moderate risk when studies relied solely on splitting the same dataset without true external validation, and High risk when no clear testing dataset evaluation was described. Reporting of confusion matrices or reconstructable performance counts was rated as Low risk when a confusion matrix or sufficient information to derive true positive (TP), false positive (FP), true negative (TN), and false negative (FN) was available, and High risk when such information was absent. Reproducibility was rated as Low risk when code or trained models were publicly accessible and High risk when neither was provided. Finally, explainability and mechanistic interpretability was rated as Low risk when studies reported feature importance, mechanistic insights, or other explainable artificial intelligence (XAI) approaches, and High risk when no explainability method was described.

## 3. Results

### 3.1. Summary of Selected Studies

**Figure 1:**
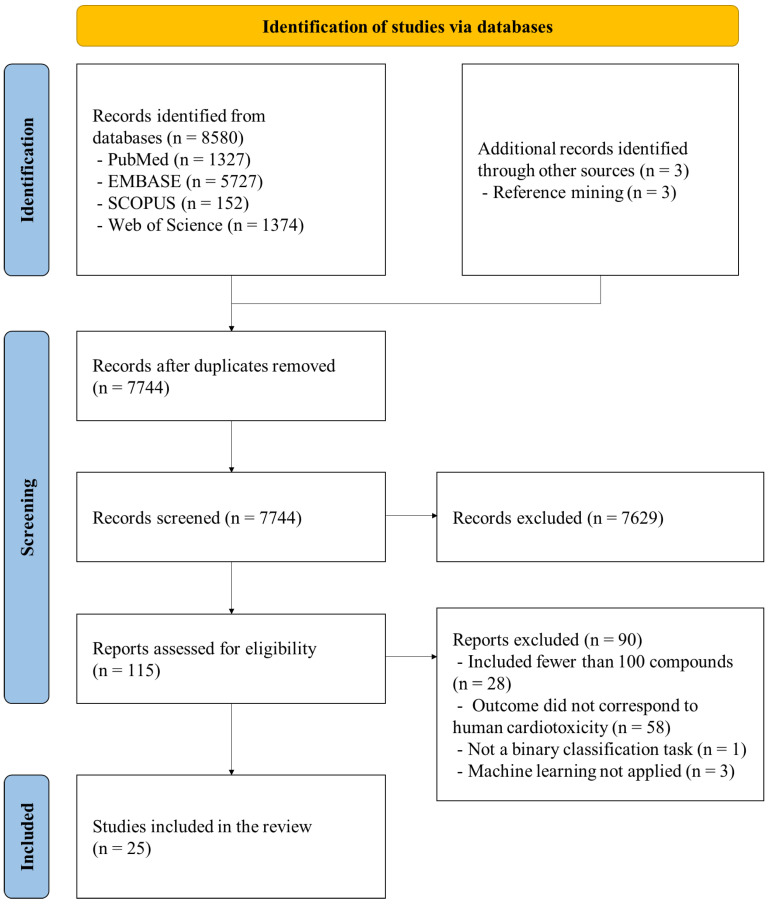
The PRISMA flow diagram for the scoping review.

The selection process is outlined in Figure 1. A total of 8580 records were initially identified through PubMed, EMBASE, SCOPUS, and Web of Science searches. Following a two-step screening process, 25 studies met the inclusion criteria and were included in the final analysis [2,11,16,17,18,19,20,21,22,23,24,25,26,27,28,29,30,31,32,33,34,35,36,37,38].

**Figure 2:**
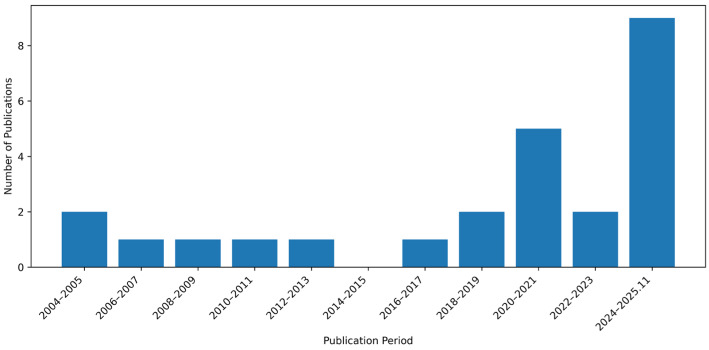
Number of publications by year range.

Table 2 presents a summary of the selected studies. These studies were published between 2004 and 2025, with the majority appearing after 2020 (Figure 2), highlighting recent advances in ML models for predicting drug-induced cardiotoxicity.

### 3.2. Summary of Databases, Selected Features, and Algorithms

Cardiotoxicity information was most commonly obtained from the SIDER (Side Effect Resource) database, which was used in 9 of the 25 studies reviewed [42]. The AZCERT website, renamed CredibleMeds (www.crediblemeds.org) in 2014, was directly used in six studies. Other data sources included OMOP (Observational Medical Outcomes Partnership) (3/25) [43], Micromedex (www.micromedexsolutions.com) (3/25), and DICTrank (Drug-Induced Cardiotoxicity Rank) (3/25) [44]. The key characteristics of these data sources are summarized in Table 3. In particular, SIDER and Micromedex provide information on a broad range of adverse drug reactions, whereas DICTrank specifically focuses on drug-induced cardiotoxicity, and CredibleMeds provides curated evidence related to TdP. The OMOP database, which provides information on both positive and negative drugs for acute myocardial infarction, was used for external validation after developing an overall adverse drug event prediction model.

Studies employed a wide range of molecular features, including linear solvation energy relationship (LSER), Mordred, RDKit, and molecular operating environment (MOE) 2D descriptors, as well as various molecular fingerprints (e.g., Molecular Access System [MACCS], Extended Connectivity Fingerprints [ECFP], PubChem, Estate). In addition, many models incorporate biological and functional features, such as drug-target protein interaction networks, gene ontology enrichment vectors, electrophysiological parameters (e.g., T_x_, T_qNet_, T_triang_), and pharmacokinetic properties like C_max_.

The most commonly used models were SVM and random forest (RF) classifiers, followed by logistic regression variants, k-nearest neighbors, and boosting models. In addition, several studies explored ensemble approaches, and more recent work has incorporated deep learning architectures, including MoLFormer-XL-CNN and attention-based graph neural networks.

### 3.3. Performance Across Cardiotoxicity Outcomes

**Figure 3:**
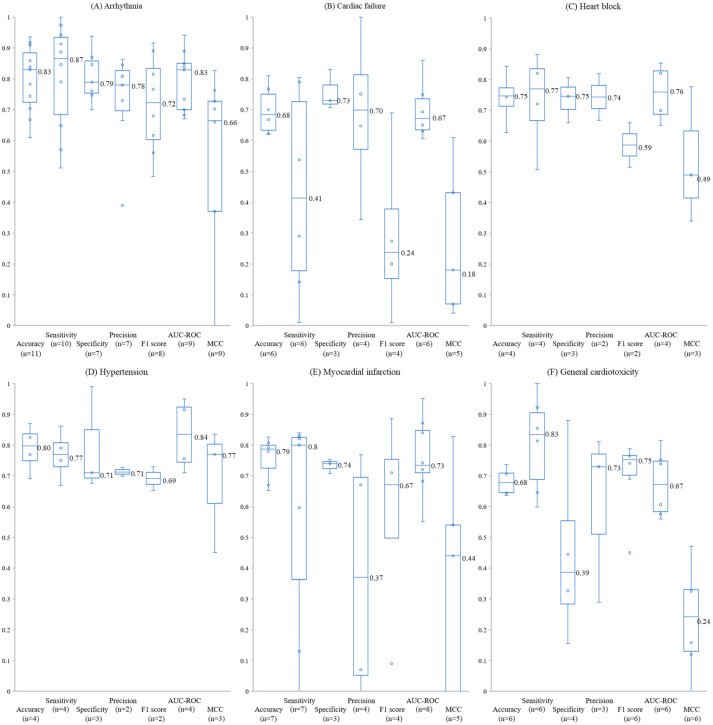
Box plots of classification performance metrics on the test or external validation set. Panels (**A**–**F**) correspond to arrhythmia, cardiac failure, heart block, hypertension, myocardial infarction, and general cardiotoxicity, respectively. Numerical values shown on the plots indicate the median for each performance metric.

Figure 3 displays box-and-whisker plots of classification metrics for six distinct cardiotoxicity endpoints, with median values annotated on each plot. The observed distributions highlight substantial variability in predictive performance across different adverse event types. However, Kruskal–Wallis tests conducted for each performance metric yielded nonsignificant *p*-values (0.091, 0.121, 0.44, 0.626, 0.249, 0.23, and 0.081 for accuracy, sensitivity, specificity, precision, F1 score, AUC–ROC, and MCC, respectively), indicating that the observed differences in medians across the six cardiotoxicity endpoints were not statistically significant.

Models tended to show relatively higher and more stable accuracy and MCC for arrhythmia and hypertension, whereas cardiac failure and general cardiotoxicity exhibited greater variability and lower central tendencies, suggesting that these phenotypes remain more challenging to model despite the absence of statistically significant differences.

#### 3.3.1. Arrhythmia

A total of 16 studies have evaluated ML models for predicting drug-induced arrhythmia (Table 4). RF models were examined by Mamoshina et al. [27], Kadioglu et al. [29], Iftkhar et al. [30], Kelleci Çelik et al. [32], and Rouen et al. [38]. These models showed test set AUC-ROC values between 0.670 and 0.941, with accuracy reported between 0.668 and 0.918. External validation results included AUC-ROC of 0.700 and accuracy ranging from 0.610 to 0.838. Notably, the model by Kadioglu et al. [29] achieved strong external validation performance, with accuracy of 0.838, sensitivity of 0.887, and specificity of 0.789.

SVM models developed by Yap et al. [16], Xue et al. [17], Bhavani et al. [18], and Yang et al. [19], which were all conducted before 2010 and typically applied to smaller datasets (e.g., 349 samples), demonstrated strong external performance with accuracy ranging from 0.910 to 0.936, although AUC-ROC was not reported. The model by Yap et al. [16] which used LSER descriptors as features, achieved particularly high sensitivity of 0.974 and specificity of 0.846.

The combined classifiers model proposed by Cai et al. [23] which integrated five ML algorithms (logistic regression, RF, k-nearest neighbors, SVM, and neural network), achieved external validation performance with an AUC-ROC of 0.734, accuracy of 0.704, sensitivity of 0.648, and specificity of 0.761.

The attention-based graph neural network (AGN) model by Vinh et al. [34], ToxBERT by He et al. [35], the MoLFormer-XL-CNN by Lin et al. [33], and the ensemble model by Li et al. [11] all demonstrated strong performance, with AUC-ROC values exceeding 0.8 on their respective test sets.

Meanwhile, models proposed by He et al. [21] and Llopis-Lorente et al. [26] reported only training or cross-validation results without independent test or external validation datasets. Such results may overestimate model performance due to overfitting and limited generalizability.

#### 3.3.2. Cardiac Failure

Six studies focused on predicting cardiac failure (Table 4). Cai et al. [23] reported that their combined classifier approach achieved an external validation AUC-ROC of 0.693 and accuracy of 0.622. The RF model by Mamoshina et al. [27] showed low external performance, with an AUC-ROC of 0.650 and F1 score of 0.010. Models proposed by Iftkhar et al. [30] and Vinh et al. [34] also demonstrated suboptimal performance in the testing dataset, with Iftkhar et al. [30] reporting an AUC-ROC of 0.630, sensitivity of 0.290, and F1 score of 0.690, while Vinh et al. [34] reported an AUC-ROC of 0.749, accuracy of 0.621, and F1 score of 0.274.

In contrast, work by Kadioglu et al. [29] demonstrated good external validation performance using a RF model that incorporated molecular features, achieving accuracy of 0.767, sensitivity of 0.804, and specificity of 0.730. Similarly, Li et al. [11] achieved strong test set performance with their ensemble model, reporting an AUC-ROC of 0.861, accuracy of 0.810, sensitivity of 0.790, and specificity of 0.830.

#### 3.3.3. Heart Block

Five studies examined models for predicting heart block (Table 4). Combined classifier approaches developed by Cai et al. [23] achieved external validation performance with an AUC-ROC of 0.699, accuracy of 0.627, sensitivity of 0.507, and specificity of 0.746. RF model developed by Kadioglu et al. [29] showed strong external validation results, achieving accuracy of 0.843, sensitivity of 0.881, and specificity of 0.806.

Other approaches, such as AGN model developed by Vinh et al. [34], demonstrated moderate performance on the testing dataset, with an AUC-ROC of 0.854, accuracy of 0.743, and F1 score of 0.515. Similarly, the ensemble model by Li et al. [11] achieved an AUC-ROC of 0.820, accuracy of 0.750, and sensitivity of 0.820 on the testing dataset, although specificity was lower at 0.660.

#### 3.3.4. Hypertension

Five studies evaluated prediction models for hypertension (Table 4). Combined classifier approaches developed by Cai et al. [23] achieved external validation performance with an AUC-ROC of 0.756, accuracy of 0.690, sensitivity of 0.669, and specificity of 0.710. RF model developed by Kadioglu et al. [29] showed strong external validation results, achieving accuracy of 0.769, sensitivity of 0.862, and specificity of 0.676.

Another RF model proposed by Iftkhar et al. [30] demonstrated good performance on the testing dataset, with an AUC-ROC of 0.710, sensitivity of 0.750, and F1 score of 0.730. AGN model proposed by Vinh et al. [34] also showed strong results on the testing dataset, with an AUC-ROC of 0.915, accuracy of 0.825, and F1 score of 0.653. Similarly, the ensemble model by Li et al. [11] achieved excellent test set performance, reporting an AUC-ROC of 0.950, accuracy of 0.870, sensitivity of 0.790, and specificity of 0.990.

#### 3.3.5. Myocardial Infarction

Nine studies investigated models for predicting myocardial infarction (Table 4). Cai et al. [23] developed a combined classifier that achieved an external validation AUC-ROC of 0.742 and accuracy of 0.652. The RF model by Kadioglu et al. [29] showed strong external validation performance, with accuracy of 0.787, sensitivity of 0.820, and specificity of 0.753. Iftkhar et al. [30] proposed a gradient boosting model that achieved testing dataset results with an AUC-ROC of 0.720, sensitivity of 0.830, and F1 score of 0.710. Similarly, Vinh et al. [34] developed an AGN model that showed testing dataset performance with an AUC-ROC of 0.872, accuracy of 0.808, and F1 score of 0.633.

The ensemble SVM model developed by Joshi et al. [28] demonstrated strong external validation performance on the OMOP dataset, with sensitivity of 0.840. The extra trees model by Wang et al. [22] also achieved high performance on the OMOP external dataset, with an AUC-ROC of 0.951, accuracy of 0.826, and F1 score of 0.886.

In contrast, the L2-regularized logistic regression model from Dey et al. [24] showed lower performance on the external dataset, with an AUC-ROC of 0.682. Similarly, the RF model developed by Mamoshina et al. [27] demonstrated limited external validation performance, with an AUC-ROC of 0.550 and accuracy of 0.670.

#### 3.3.6. General Cardiotoxicity

Seven studies focused on general cardiotoxicity (Table 4). The RF model developed by Seal et al. [2] demonstrated good performance on the testing dataset, with an AUC-ROC of 0.691, accuracy of 0.763, sensitivity of 0.646, and specificity of 0.880. Another RF model by Mamoshina et al. [27] showed moderate performance on the testing dataset with an AUC-ROC of approximately 0.739, but lower performance on the external dataset, with an AUC-ROC of 0.560, accuracy of 0.710, and F1 score of 0.450. Qu et al. [37] developed logistic regression and extreme gradient boosting (XGBoost) models, achieving AUC-ROC values from 0.576 to 0.606 and accuracy from 0.637 to 0.644 on the testing dataset, suggesting limited predictive performance. In addition, the model proposed by Li et al. [36] demonstrated moderate performance, achieving an AUC-ROC of 0.751 on the testing dataset.

Amano et al. [25], Ye et al. [31], and Huang et al. [20] also developed models with cross-validation AUC-ROC values ranging from 0.668 to 0.830; however, they did not report performance on independent testing or external validation datasets.

### 3.4. Study Quality

Table 5 presents a summary of the study quality and the risk-of-bias assessment for the included studies. Because data-source availability was an inclusion criterion, studies were rated as low risk in the provenance domain, except for Kadioglu et al. [29]. Although this study reported the source of its training data, it did not disclose the source of the data used for external validation.

A total of 9 studies conducted true external validation. Among these, the study by He et al. [35] was excluded from Table 4 because it assessed performance using proportional reporting ratio (PRR) values derived from the U.S. Food and Drug Administration (FDA) Adverse Event Reporting System (FAERS) dataset. In addition, 8 studies generated a test dataset by splitting their original dataset.

Regarding the confusion matrix, only 5 of the 25 included studies explicitly reported one. In 4 additional studies, a confusion matrix could be reconstructed from the reported total number of data points, accuracy, sensitivity, and precision. Code availability was confirmed for 12 of the 25 studies, and most studies (19 of 25) performed explainability analyses.

Overall, the study by Yang et al. [19] was rated as Low risk in only one of the six assessment domains. Similarly, the studies by He et al. [21], Kadioglu et al. [29], and Vinh et al. [34] met the Low risk criteria in only two domains, indicating that caution is warranted when interpreting and accepting the findings of these studies.

## 4. Discussion

This scoping review systematically examined 25 studies applying ML models to predict drug-induced cardiotoxicity, summarizing the data sources used, the types of features and ML algorithms considered, and the predictive performance reported for specific outcomes. Most studies relied on structured databases such as SIDER, CredibleMeds, OMOP, and FDA labeling information to identify drug–adverse event associations. Features ranged from molecular descriptors and fingerprints to, less frequently, target-based and transcriptomic data. Reported model performance varied across outcomes, with some studies demonstrating externally validated AUC-ROC values exceeding 0.70 or classification accuracy above 0.75 for endpoints such as arrhythmias, cardiac failure, heart block, hypertension, and myocardial infarction.

Whereas ML approaches for predicting hERG inhibition have been extensively studied since before 2010 [45], our review reveals a marked increase in studies addressing human cardiotoxicity outcomes beginning in 2020. Information on drug-induced cardiotoxicity was primarily collected from the SIDER database. However, SIDER has several limitations. First, SIDER provides limited information on negative examples, as any unmentioned drug–adverse event pair is generally treated as a negative case, which can introduce labeling bias. Importantly, such unlabeled pairs may not represent true negatives, and treating them as confirmed negative cases can lead to misclassification and potentially inflate model specificity. This issue is not unique to SIDER but is inherent to most cardiotoxicity resources which similarly lack explicit confirmation of true negative cases. Second, it is a static database last updated around 2015, and therefore may not capture newly approved drugs or recent safety warnings. More recently, resources such as the FDA labeling-based DICTrank [44] and the DIQTA [46] have been released and used to support ML research on cardiotoxicity prediction [2,33,37]. These newer resources offer more up-to-date, structured information, helping to address some of the limitations of older datasets like SIDER. As such datasets continue to expand and improve, they are expected to facilitate more generalizable ML models.

SVM and RF were the most commonly used algorithms in the studies included in this scoping review. Notably, studies by Yap et al. [16] and Bhavani et al. [18] using SVM, as well as those by Kadioglu et al. [29], Seal et al. [2], and Kelleci Çelik et al. [32] employing RF, demonstrated excellent predictive performance. SVM and RF are also among the most widely used algorithms for predicting various types of toxicity, including hERG blockade, hepatotoxicity, cardiotoxicity, and carcinogenicity [45,47]. This strong classification performance can be attributed to the ability of SVM to effectively handle high-dimensional feature spaces and identify complex decision boundaries, while RF offers robustness to noise, handles nonlinear relationships well, and reduces overfitting through ensemble learning [47].

Despite their effectiveness, ongoing algorithmic advancements remain essential. Recent studies have demonstrated improved performance by integrating Isometric Stratified Ensembles with XGBoost for highly imbalanced datasets, incorporating large language models for embedding feature computation, and applying Graph Neural Networks to predict hERG blockade [48,49,50]. Future research should investigate integrating deep learning architectures, developing more sophisticated ensemble frameworks, and employing automated ML techniques to optimize model performance and generalizability.

In this scoping review, features such as target proteins and transcriptional profiles were considered in some studies. However, several included studies also demonstrated that models relying solely on molecular features could achieve strong predictive performance. While our review focused on cardiotoxicity, it is worth noting that research in other toxicity domains has similarly highlighted the power of molecular descriptors. A particularly strong example is the work by Mayr et al., which, won the Tox21 competition with their DeepTox model [51]. Their approach leveraged thousands of physicochemical descriptors, widely used fingerprints such as PubChem substructure fingerprints, ECFP, and MACCS Keys [51]. Despite these advancements, optimizing feature selection and integration to enhance model performance remains a significant challenge. Carefully selecting biologically relevant features that correlate strongly with cardiotoxic outcomes is crucial for improving both predictive performance and interpretability, which are fundamental for clinical and regulatory adoption [52]. Future research should continue to refine feature selection processes to ensure that integrated features adequately capture the complexity of cardiotoxicity while minimizing noise and redundancy in the models. Moreover, it is crucial to recognize that these feature-based approaches have been developed for small molecules, not for biologics, such as peptides, antibody-drug conjugates, or gene therapies.

In addition to optimizing feature selection, future research should explore the integration of mechanistic insights at the individual compound level to better capture the biological diversity of drug-induced cardiotoxicity. Different drugs may induce cardiotoxic effects through distinct molecular pathways, such as mitochondrial dysfunction, ion channel interference, and inflammatory signaling [10,53]. Developing mechanism-informed machine learning models that incorporate such pharmacological and toxicological knowledge could enhance both predictive performance and interpretability.

Furthermore, personalized, integrated ML models that incorporate drug-specific cardiotoxicity predictions alongside real-world clinical data is proposed. By utilizing electronic health records (EHRs), including patient medication history, comorbidities, and longitudinal cardiovascular outcomes, these models could enable individual-level cardiotoxicity risk prediction for patients actively receiving the drug of interest. Such an approach aligns with the goals of precision medicine and has been demonstrated to be feasible in recent studies integrating multi-omic data, causal inference networks, and EHR-based phenotyping to predict adverse drug reactions [54]. Expanding this approach to cardiotoxicity prediction could be a valuable step forward for both drug safety monitoring and clinical decision-making.

Regulatory considerations represent another essential component of the deployment pathway. For cardiotoxicity specifically, established frameworks such as the Comprehensive in vitro Proarrhythmia Assay (CiPA) initiative and the ICH S7B guidelines [55] provide valuable standards for evaluating proarrhythmic risk. Aligning ML validation strategies with these frameworks would help ensure that model outputs are consistent with accepted toxicological and pharmacological assessments. Moreover, although the U.S. FDA guidance on AI/ML-enabled Software as a Medical Device (SaMD) is primarily directed toward medical devices rather than drug safety models [56], the regulatory principles it outlines, including appropriate dataset governance, continuous performance monitoring, transparency in model updates, and clear documentation of intended use, provide a useful framework for guiding the future clinical translation of ML-based cardiotoxicity predictors. Incorporating these principles from the early stages of model development can enhance regulatory alignment and ultimately support safer and more reliable implementation.

This review has several important considerations. First, although a meta-analysis with a summary receiver operating characteristic curve could have provided a more quantitative synthesis of predictive model performance, this was not feasible due to inconsistent reporting across studies. Many included studies did not provide essential metrics such as TP, FP, TN, and FN, limiting the ability to calculate pooled performance estimates. As a result, we focused on qualitatively describing reported outcomes and trends, highlighting key findings across studies. To facilitate more robust and comparable future syntheses, we encourage researchers to consistently report these fundamental performance metrics in their publications. Second, substantial heterogeneity across datasets, feature types, and algorithms hampered direct comparison of model performance. Third, many studies showed High risk in the areas of imbalance, external validation, confusion matrix reporting, and code availability, indicating notable concerns regarding methodological quality and reproducibility. Moreover, most available cardiotoxicity resources lack confirmed true negative labels, introducing potential labeling noise and posing a risk of inflated specificity due to uncertain negative-class assignments. Finally, the rapid evolution of machine learning methods means that newer and potentially more effective models may have emerged since the completion of this review.

## 5. Conclusions

In conclusion, this scoping review summarized ML models developed to predict human drug-induced cardiotoxicity such as arrhythmia, cardiac failure, heart block, hypertension, and myocardial infarction. ML models, particularly those using SVM and RF algorithms, demonstrated promising predictive performance in several studies. However, challenges remain, including the lack of consistent external validation and reliance on static or outdated data sources. Future research should prioritize the development of larger, more comprehensive, and up-to-date datasets, systematic external validation, and integration of diverse and biologically meaningful features to improve model generalizability, interpretability, and clinical utility.

## Figures and Tables

**Table 1:** Search queries for PubMed, Embase, Web of Science, and SCOPUS.

Source	Search Query	Date Range	Hits
PubMed	(“Cardiotoxicity”[Mesh] AND “Myocardial Infarction”[Mesh] OR “Heart Valves” [Mesh] OR “Atrial Fibrillation”[Mesh] OR “Arrhythmias, Cardiac” [Mesh] OR “Long QT Syndrome”[Mesh] OR “Torsades de Pointes”[Mesh] OR “Atrial Flutter”[Mesh]) AND (“Artificial Intelligence”[Mesh] OR artificial intelligence OR machine learning OR deep learning) AND (drug OR medication OR pharmaceutic *)	Up to 25 November 2025	1327
Embase	#1. ‘cardiotoxicity’/exp OR cardiotoxicity #2. ‘heart infarction’/exp OR ‘heart infarction’ #3. ‘myocardial infarction’ #4. ‘heart valve’/exp OR ‘heart valve’ #5 ‘atrial fibrillation’/exp OR ‘atrial fibrillation’ #6. ‘heart arrhythmia’/exp OR ‘heart arrhythmia’ #7. ‘qt prolongation’/exp OR ‘qt prolongation’ #8. ‘torsade de pointes’/exp OR ‘torsade de pointes’ #9. ‘heart atrium flutter’/exp OR ‘heart atrium flutter’ #10. ‘atrial flutter’ #11. #1 OR #2 OR #3 OR #4 OR #5 OR #6 OR #7 OR #8 OR #9 OR #10 #12. ‘drug’/exp OR drug #13. Medication #14. pharmaceutic * #15. #12 OR #13 OR #14 #16. ‘artificial intelligence’/exp OR ‘artificial intelligence’ #17. ‘machine learning’/exp OR ‘machine learning’ #18. ‘deep learning’/exp OR ‘deep learning’ #19. #16 OR #17 OR #18 #20. #11 AND #15 AND #19	Up to 25 November 2025	5727
Web of Science	(TS = (Cardiotoxicity) OR TS = (Myocardial Infarction) OR TS = (Heart Valves) OR TS = (Atrial Fibrillation) OR TS = (arrhythmia) OR TS = (qt prolongation) OR TS = (Torsades de Pointes) OR TS = (Atrial Flutter)) AND (ALL = (artificial intelligence) OR ALL = (machine learning) OR ALL = (deep learning)) AND (ALL = (drug) OR ALL = (medication) OR ALL = (pharmaceutic *))	Up to 25 November 2025	1374
SCOPUS	((TITLE-ABS-KEY(Cardiotoxicity)) OR (TITLE-ABS-KEY(Myocardial Infarction)) OR (TITLE-ABS-KEY(Heart Valves)) OR (TITLE-ABS-KEY (Atrial Fibrillation)) AND (TITLE-ABS-KEY(arrhythmia)) AND (TITLE-ABS-KEY(qt prolongation)) OR (TITLE-ABS-KEY(Torsades de Pointes)) OR (TITLE-ABS-KEY(Atrial Flutter))) AND ((ALL(artificial intelligence)) OR (ALL(machine learning)) OR (ALL(deep learning))) AND ((ALL(drug)) OR (ALL(medication)) OR (ALL(pharmaceutic *)))	Up to 25 November 2025	152

The asterisk (*) denotes a functional symbol used as-is in databases such as PubMed for search and annotation purposes.

**Table 2:** Summary of studies on predictive models for drug-induced cardiotoxicity.

Reference	Year	Cardiotoxicity Database	Included Features		Outcome	Algorithms	Summary
			Molecular Features	Other Features			
[16]	2004	AZCERT, Micromedex *, Drug Information Handbook *, Meyler’s Side Effects of Drugs *, De ponti et al. [39] *, AHFS Drug Information *	LSER descriptors		Arrhythmia (TdP)	SVM PNN kNN C4.5 decision tree	SVM approach using LSER descriptors achieved prediction accuracies of 97.4% for TdP-causing agents and 84.6% for non-TdP-causing agents.
[17]	2004	AZCERT, Micromedex, Drug Information Handbook, Meyler’s Side Effects of Drugs, AHFS Drug Information	Molecular descriptors		Arrhythmia (TdP)	SVM	The SVM model using recursive feature elimination achieved accuracies of 66.8% for TdP+ and 89.3% for TdP−.
[18]	2006	Yap et al. [16]	Number of instances of substructures, Euclidean distances		Arrhythmia (TdP)	SVM	Combining weighted instances with the Euclidean distance measure improved prediction accuracy to 93.59%.
[19]	2009	Xue et al. [17]	Molecular descriptors (PCLI-ENT online program)		Arrhythmia (TdP)	SVM	The SVM model using a genetic algorithm and the conjugate gradient method achieved accuracies of 64.7% for TdP+ and 92.8% for TdP−.
[20]	2011	SIDER		Drug target expanding protein-protein interaction networks network	General cardiotoxicity	SVM LR	Incorporating protein-protein interaction networks and gene ontology annotations significantly improved SVM-based cardiotoxicity prediction, achieving a median AUC of 0.771, accuracy of 0.675, sensitivity of 0.632, and specificity of 0.789.
[21]	2012	Micromedex, AZCERT	Molecular descriptors (PaDEL-descriptor version 2.7)		Arrhythmia (TdP)	OCSVM OCLOF OCPD Their ensemble model	A final ensemble model was constructed based on selected base models and it had sensitivity and specificity value of 78.4% and 90% respectively.
[22]	2016	SIDER, Offsides, OMOP *	Molecular fingerprints (MACCS)	Gene ontology enrichment vector from gene expression (LINCS L1000 dataset)	Myocardial infraction	RF SVM L1-regularized LR NB kNN Extra tree	Extra tree classifiers show improved AUC-ROC, F1-score, and accuracy for acute myocardial infarction when combining gene ontology enrichment vectors with chemical structure features. Available at: http://maayanlab.net/SEP-L1000/ (accessed on 10 December 2025)
[23]	2018	Comparative Toxicogenomics Database, SIDER, MetaADEDB, Offsides *	2D descriptors (MOE 2010 software), molecular fingerprints (MACCS, Estate, Pubchem, substructure fingerprint)		Arrhythmia, cardiac failure, heart block, hypertension, myocardial infarction	kNN LR RF SVM Their combined classifier	Combined classifiers had 87% success rate in predicting cardiotoxicity.
[24]	2018	SIDER, OMOP *	Molecular fingerprint (ECFP6)		Myocardial infarction	L2-regularized LR	The ECFP6 circular fingerprint method outperformed the neural fingerprint method in predicting acute myocardial infarction.
[25]	2020	SIDER		Known and newly estimated target proteins	General cardiotoxicity	L1-regularized LR	Chemical-protein interaction-base model performed better than or comparable to the chemical structure-based model.
[26]	2020	CredibleMeds		T_x_ (drug concentration prolonging action potential by 10%), T_qNet_ (net charge carried by ionic currents), T_triang_ (triangulation of drug concentration over control)	Arrhythmia (TdP)	Decision tree	Combining T_x_, T_qNet_, and T_traing_ in a decision tree classifier improved prediction accuracy to 94.5%. Available at: https://riunet.upv.es/handle/10251/136919 (accessed on 10 December 2025)
[27]	2020	SIDER	Molecular weight, partition coefficient, atomic polarizabilities, topological polar surface area, polar surface area expressed as a ratio to molecular size, Ghose-Crippen LogKow, molar refractivity, molecular fingerprints (Estate)	Transcriptional profiles of landmark genes	Arrhythmia, cardiac failure, myocardial disorders, general cardiotoxicity	RF GB CatBoost Elastic net	The chain of RF classifiers achieved the best performance, with an average AUC of 0.79 on validation and 0.66 on testing across all cardiotoxicity forms.
[28]	2021	SIDER, OMOP *	Atom-level features (type of atoms, degree of the atom, number of hydrogen atoms connected, valence of the atom), bond-level features (bond type, conjugation, ring presence)		Myocardial infarction	Ensemble SVM	Ensembled SVM based approach outperformed the competing method in predicting acute myocardial infraction, achieving a sensitivity of 0.84.
[29]	2021	DrugBank	LogP, drug likeness, amines, ligand efficiency, alkyl-amines, aromatic nitrogens, basic nitrogens		Arrhythmia, cardiac failure, heart block, hypertension, myocardial infarction	RF SVM kNN NB AdaBoost	RF demonstrated optimal performance, achieving AUC scores above 0.830 across all five cardiotoxicity indications.
[30]	2022	Karim et al. [40], Cai et al. [23], Munawar et al. [41]	General molecular descriptors from RDKit, graph-based signatureso model geometry and physicochemical properties, toxicophore matchings via substructure search, molecular fingerprints		Arrhythmia, cardiac failure, heart block, hypertension, myocardial infarction	RF GB XGBoost	The models achieved AUCs of up to 0.898 under 5-fold cross-validation and generalizable performance (up to 0.951) in the blind test sets. Available at: https://biosig.lab.uq.edu.au/cardiotoxcsm (accessed on 10 December 2025)
[31]	2022	ChemIDplus, Pharmapendium^®^, CDER, NCTR, Enzo Life Sciences cardiotoxicity library, SIDER	Extended-connectivity fingerprints (ECFP4)		General cardiotoxicity	RF NB XGBoost SVM	Chemical structure-based models showed good predictive power for DICT (AUC-ROC = 0.83 ± 0.03), while Tox21 assay data performed only slightly better than random.
[32]	2024	CredibleMeds	Molecular descriptors (PaDEL-descriptor)		Arrhythmia (TdP)	SVM XGBoost RF CatBoost	RF algorithm achieved the best overall performance across accuracy rate, sensitivity, MCC, and F1 score.
[33]	2024	DIQTA	SMILES		Arrhythmia (QTc prolongation)	MoLFormer-XL-CNN	MoLFormer-XL-CNN model, using SMILES as input, outperformed conventional models in predicting DIQT.
[2]	2024	DICTrank, SIDER	Mordred descriptors, ECFP4 fingerprints	Mechanism of action, CELLSCAPE target prediction dataset, cell painting, gene expression, gene ontology, C_max_	General cardiotoxicity	RF	Models based on physicochemical properties achieved high predictive accuracy (AUCPR = 0.93). Available at: https://broad.io/DICTrank_Predictor (accessed on 10 December 2025)
[34]	2024	Iftkhar et al. [30]	Atomic, degree, IsCharge, orbital hybridization, IsAromatic, Caln-Ingold-Prelog priority, chirality features		Arrhythmia, cardiac failure, heart block, hypertension, myocardial infarction	AGN	The simplified attention-based graph neural network outperformed traditional ML and advanced graph-based models.
[35]	2025	DIQTA	SMILES		Arrhythmia (QTc prolongation)	BERT	ToxBERT achieved AUC-ROC of 0.839 for predicting drug-induced QT prolongation.
[11]	2025	Cai et al. [23], Iftkhar et al. [30]	Molecular descriptors (Online Chemical Modeling Environment platform)		Arrhythmia, cardiac failure, heart block, hypertension, myocardial infarction	Ensemble model of 7 machine learning and 5 deep learning models	A total of 110 predictive models were constructed for each cardiotoxicity endpoint and the consensus models consistently outperformed individual models. Available at: https://ochem.eu/article/166881 (accessed on 10 December 2025)
[36]	2025	DICTrank	Chemical descriptors (Mordred)	GPT-4o–generated pharmacology-and-toxicity summary embedding	General cardiotoxicity	LR kNN RF SVM XGBoost	Quantitative Knowledge & Structure-Activity Relationships model consistently outperformed QSAR model
[37]	2025	DICTrank	Molecular descriptors (Mold2 software)		General cardiotoxicity	LR kNN SVM RF XGBoost	LR and XGBoost achieved the best results with DICTrank.
[38]	2025	CredibleMeds	Molecular descriptors	Drug interactions with the hERG, Na_V_1.5, Ca_V_1.2 channels	Arrhythmia (TdP)	LR RF XGBoost Neural net	RF model using computed drug binding affinities for hERG and Ca_V_1.2 channels achieved 94% overall model accuracy.

* External validation dataset. Abbreviations: AGN, attention-based graph neural network; AUC-ROC, area under the receiver operating characteristic curve; AUCPR, area under the precision recall curve; BERT, bidirectional encoder representations from transformers; CDER, Center for Drug Evaluation and Research; CNN, convolutional neural network; DICTrank, Drug-Induced Cardiotoxicity Rank; DIQTA, Drug-Induced QT Prolongation Atlas; ECFP, Extended Connectivity Fingerprints; GB, gradient boosting; hERG, human Ether-à-go-go-Related Gene; kNN, k-nearest neighbors; LINCS, Library of Integrated Network-based Cellular Signatures; LR, logistic regression; LSER, linear solvation energy relationship; MACCS, Molecular Access System; MOE, molecular operating environment; NB, Naïve Bayes; NCTR, National Center for Toxicological Research; OCLOF, One-Class Local Outlier Factor; OCPD, One-Class Probability Density; OCSVM, One-Class Support Vector Machine; OMOP, Observational Medical Outcomes Partnership; PNN, probabilistic neural network; RF, random forest; SIDER, Side Effect Resource; SMILES, Simplified Molecular Input Line Entry System; SVM, support vector machine; TdP, Torsades de Pointes; XGBoost, extreme gradient boosting.

**Table 3:** Comparative characteristics of database.

Database	Last Update	Evidence	Label	Noise/Limitation	ML Suitability
SIDER	21 October 2015	Drug labels	Positive label-only: drug–adverse event pairs explicitly reported in the labels.	Negative label: positive label-only. Update: outdated. Evidence reliability: text-extracted AEs may be ambiguous or noisy. Labeling variability introduces noise.	Useful for large-scale drug–AE association modeling. Freely available for download.
CredibleMeds	Updated regularly	Drug labels, literature, case reports	Positive label: drugs classified into Known, Possible, and Conditional risk of TdP based on expert review. Negative label: therapeutic options that are not included on the QT drug list.	Negative label: non-listed drugs are not guaranteed safe. Accessibility: limited scope (TdP) and login required. Evidence reliability: Relies on available literature and case reports (publication bias).	High-quality expert labels valuable for supervised classification of QT/TdP risk; limited size but strong signal.
DICTrank	November 2023	Drug labels	Four-level label: drugs classified into Most-, Less-, Ambiguous-, or No-DICT-Concern based on FDA labeling sections and the severity of reported cardiotoxicity. Most-DICT-Concern: withdrawn drugs or those with BW-level or severe/moderate DICT in WP. Less-DICT-Concern: mild DICT in WP or DICT mentioned in AR/Overdosage. Ambiguous-DICT-Concern: DICT keywords present only in special clinical contexts. No-DICT-Concern: no DICT-related information in any labeling section.	Negative label: “No-DICT-Concern” only reflects absence in labeling, not true safety. Update: static snapshot. Evidence reliability: rule-based classification may misinterpret context. Labeling variability introduces noise.	Structured multi-level toxicity labels good for ordinal classification. Freely available for download.
Micromedex	Updated regularly	Drug labels, literature	Positive label-only: drug–adverse event pairs explicitly reported in the labels or literature.	Negative label: positive label-only. Accessibility: subscription-based Evidence reliability: mixed sources lead to variable consistency. Variable evidence depth.	Good for training models requiring clinically validated AE signals.
OMOP	October 2013	Drug labels, literature	Positive label: drugs with MI-related evidence from labels and the literature, with no conflicting studies. Negative label: drugs without MI-related terms in labels, without supporting literature.	Negative label: absence of MI-related evidence does not ensure no risk. Update: outdated Evidence reliability: publication bias.	Positive/negative AE associations useful for binary classification and validation tasks.

Abbreviations: AE, adverse event; AR, adverse reactions; BW, boxed warning; DICT, drug-induced cardiotoxicity; MI, myocardial infarction; TdP, torsade de pointes; WP, warnings and precautions.

**Table 4:** Summary of best-performing model performance metrics by dataset type.

Outcome	Model	Number of Compounds				Performance Metrics							Reference
		Total	Train	Test	External	Accuracy	Sensitivity	Specificity	Precision	F1 Score	AUC-ROC	MCC	
Arrhythmia	AGN	1496	1346	150	-	[b] 0.632 [c] 0.744	-	-	-	[b] 0.573 [c] 0.484	[b] 0.692 [c] 0.850	[b] 0.271 [c] 0.727	[34]
	BERT	251	-	-	-	[c] 0.783	[c] 0.913	[c] 0.700	[c] 0.664	[c] 0.766	[c] 0.839	[c] 0.664	[35]
	CNN	255	-	-	-	[c] 0.859	[c] 0.942	[c] 0.747	[c] 0.845	[c] 0.891	[c] 0.829	[c] 0.702	[33]
	Decision tree	109	109	-	-	[b] 0.945	[b] 0.940	[b] 0.950	-	-	-	-	[26]
	Ensemble model	260	260	-	-	[a] 0.912 [b] 0.856	[a] 0.889 [b] 0.784	[a] 0.928 [b] 0.900	-	-	[a] 0.932 [b] 0.825	[a] 0.817 [b] 0.692	[21]
		1592	1450	-	142	[b] 0.717 [d] 0.704	[b] 0.712 [d] 0.648	[b] 0.723 [d] 0.761	[b] 0.720 [d] 0.730	-	[b] 0.784 [d] 0.734	-	[23]
		2169	1743	426	-	[c] 0.830	[c] 0.790	[c] 0.870	-	-	[c] 0.890	[c] 0.660	[11]
	RF	408	291	66	51	[b] 0.735 [c] 0.668 [d] 0.610	[b] 0.596 [c] 0.511 [d] 1.000	-	[b] 0.860 [c] 0.780 [d] 0.390	[b] 0.704 [c] 0.617 [d] 0.560	[b] 0.880 [c] 0.683 [d] 0.700	[b] 0.504 [c] 0.370 [d] 0	[27]
		1594	1451	-	143	[a] 0.770 [d] 0.838	[a] 0.775 [d] 0.887	[a] 0.764 [d] 0.789	[a] 0.767 [d] 0.808	-	[a] 0.849	-	[29]
		1568	1410	158	-	-	[b] 0.630 [c] 0.570	-	-	[b] 0.700 [c] 0.680	[b] 0.710 [c] 0.670	[b] 0.400 [c] 0.370	[30]
		141	98	43	-	[b] 0.837	[b] 0.963	[b] 0.625	[b] 0.625	[b] 0.758	-	[b] 0.651	[32]
		300	-	-	-	[c] 0.918	[c] 0.846	[c] 0.938	-	[c] 0.815	[c] 0.941	[c] 0.763	[38]
	SVM	349	271	-	78	[d] 0.910	[d] 0.974	[d] 0.846	[d] 0.864	[d] 0.916	-	[d] 0.827	[16]
		361	361	-	-	[b] 0.839	[b] 0.668	[b] 0.893	-	-	-	[b] 0.560	[17]
		349	271	-	78	[d] 0.936	-	-	-	-	-	-	[18]
		361	361	-	-	[b] 0.861	[b] 0.647	[b] 0.928	-	-	-	-	[19]
Cardiac Failure	AGN	1096	986	110	-	[b] 0.517 [c] 0.621	-	-	-	[b] 0.187 [c] 0.274	[b] 0.627 [c] 0.749	[b] 0.049 [c] 0.431	[34]
	Ensemble model	1170	630	-	540	[b] 0.711 [d] 0.622	[b] 0.686 [d] 0.537	[b] 0.737 [d] 0.707	[b] 0.722 [d] 0.647	-	[b] 0.785 [d] 0.693	-	[23]
		1201	1066	135	-	[c] 0.810	[c] 0.790	[c] 0.830	-	-	[c] 0.860	[c] 0.610	[11]
	RF	408	291	66	51	[b] 0.676 [c] 0.668 [d] 0.700	[b] 0.484 [c] 0.141 [d] 0.010	-	[b] 0.583 [c] 0.344 [d] 1.000	[b] 0.529 [c] 0.200 [d] 0.010	[b] 0.787 [c] 0.607 [d] 0.650	[b] 0.288 [c] 0.040 [d] 0.070	[27]
		1164	626	-	538	[a] 0.755 [d] 0.767	[a] 0.768 [d] 0.804	[a] 0.742 [d] 0.730	[a] 0.752 [d] 0.751	-	[a] 0.831	-	[29]
	XGBoost	1153	1037	116	-	-	[b] 0.350 [c] 0.290	-	-	[b] 0.730 [c] 0.690	[b] 0.610 [c] 0.630	[b] 0.290 [c] 0.180	[30]
Heart Block	AGN	883	794	89	-	[b] 0.651 [c] 0.743	-	-	-	[b] 0.656 [c] 0.515	[b] 0.708 [c] 0.854	[b] 0.306 [c] 0.777	[34]
	Ensemble model	946	544	-	402	[b] 0.767 [d] 0.627	[b] 0.721 [d] 0.507	[b] 0.813 [d] 0.746	[b] 0.794 [d] 0.667	-	[b] 0.842 [d] 0.699	-	[23]
		1488	1177	311	-	[c] 0.750	[c] 0.820	[c] 0.660	-	-	[c] 0.820	[c] 0.490	[11]
	RF	948	545	-	403	[a] 0.781 [d] 0.843	[a] 0.783 [d] 0.881	[a] 0.779 [d] 0.806	[a] 0.780 [d] 0.819	-	[a] 0.869	-	[29]
		932	838	94	-	-	[b] 0.750 [c] 0.720	-	-	[b] 0.730 [c] 0.660	[b] 0.720 [c] 0.650	[b] 0.460 [c] 0.340	[30]
Hypertension	AGN	1311	1179	132	-	[b] 0.622 [c] 0.825	-	-	-	[b] 0.605 [c] 0.653	[b] 0.676 [c] 0.915	[b] 0.247 [c] 0.835	[34]
	Ensemble model	1452	1162	-	290	[b] 0.739 [d] 0.690	[b] 0.750 [d] 0.669	[b] 0.728 [d] 0.710	[b] 0.734 [d] 0.698	-	[b] 0.800 [d] 0.756	-	[23]
		2228	1787	441	-	[c] 0.870	[c] 0.790	[c] 0.990	-	-	[c] 0.950	[c] 0.770	[11]
	RF	1454	1163	-	291	[a] 0.781 [d] 0.769	[a] 0.795 [d] 0.862	[a] 0.766 [d] 0.676	[a] 0.773 [d] 0.727	-	[a] 0.854	-	[29]
		1374	1236	138	-	-	[b] 0.780 [c] 0.750	-	-	[b] 0.720 [c] 0.730	[b] 0.710 [c] 0.710	[b] 0.450 [c] 0.450	[30]
Myocardial Infarction	AGN	773	695	78	-	[b] 0.655 [c] 0.808	-	-	-	[b] 0.646 [c] 0.633	[b] 0.718 [c] 0.872	[b] 0.315 [c] 0.828	[34]
	Ensemble model	816	638	-	178	[b] 0.727 [d] 0.652	[b] 0.690 [d] 0.596	[b] 0.765 [d] 0.708	[b] 0.746 [d] 0.671	-	[b] 0.790 [d] 0.742	-	[23]
		1602	1430	-	172	-	[d] 0.840	-	-	-	-	-	[28]
		1277	1048	229	-	[c] 0.780	[c] 0.800	[c] 0.740	-	-	[c] 0.840	[c] 0.540	[11]
	Extra Tree	20,413	-	-	60	[d] 0.826	-	-	-	[d] 0.886	[d] 0.951	-	[22]
	GB	803	722	81	-	-	[b] 0.690 [c] 0.830	-	-	[b] 0.660 [c] 0.710	[b] 0.690 [c] 0.720	[b] 0.340 [c] 0.440	[30]
	LR	1602	1430	-	172	-	-	-	-	-	[d] 0.682	-	[24]
	RF	818	639	-	179	[a] 0.762 [d] 0.787	[a] 0.765 [d] 0.820	[a] 0.759 [d] 0.753	[a] 0.760 [d] 0.768	-	[a] 0.834	-	[29]
		408	291	66	51	[b] 0.762 [c] 0.792 [d] 0.670	[b] 0.315 [c] 0 [d] 0.130	-	[b] 0.944 [c] 0 [d] 0.070	[b] 0.472 [c] NaN [d] 0.090	[b] 0.875 [c] 0.726 [d] 0.550	[b] 0.457 [c] −0.077 [d] −0.090	[27]
General cardiotoxicity	LR	1320	-	-	-	[b] 0.864	[b] 0.490	[b] 0.898	-	-	[b] 0.668	-	[25]
		924	621	303	-	[b] 0.805 [c] 0.706	[b] 0.860 [c] 0.855	[b] 0.610 [c] 0.445	[b] 0.888 [c] 0.730	[b] 0.873 [c] 0.788	[c] 0.751	[b] 0.455 [c] 0.332	[36]
		924	621	303	-	[b] 0.725 [c] 0.637	[b] 0.773 [c] 0.813	[b] 0.554 [c] 0.327	-	[b] 0.814 [c] 0.741	[b] 0.721 [c] 0.576	[b] 0.297 [c] 0.159	[37]
	NB	646	-	-	-	-	-	-	-	-	[b] 0.830	-	[31]
	RF	408	291	66	51	[b] 0.608 [c] 0.649 [d] 0.710	[b] 0.465 [c] 0.599 [d] 1.000	-	[b] 0.626 [c] 0.811 [d] 0.290	[b] 0.534 [c] 0.689 [d] 0.450	[b] 0.627 [c] 0.739 [d] 0.560	[b] 0.215 [c] 0.326 [d] 0	[27]
		1566	1476	90	-	[c] 0.763	[c] 0.646	[c] 0.880	-	[c] 0.764	[b] 0.691 [c] 0.814	[c] 0.471	[2]
	SVM	877	-	-	-	[b] 0.654	[b] 0.800	[b] 0.583	-	-	[b] 0.736	-	[20]
	XGBoost	924	621	303	-	[b] 0.796 [c] 0.644	[b] 0.928 [c] 0.922	[b] 0.328 [c] 0.155	-	[b] 0.877 [c] 0.767	[b] 0.782 [c] 0.606	[b] 0.319 [c] 0.120	[37]

[a] Training dataset, [b] Cross-validation, [c] Testing dataset, [d] External validation. Abbreviations: AGN, attention-based graph neural network; AUC-ROC, area under the receiver operating characteristic curve; BERT, Bidirectional Encoder Representations from Transformers; CNN, convolutional neural network; GB, gradient boosting; LR, logistic regression; MCC, Matthews correlation coefficient; NB, Naïve Bayes; RF, random forest; SVM, support vector machine; XGBoost, extreme gradient boosting.

**Table 5:** Summary of study quality and risk-of-bias assessment for included studies.

Reference	Provenance	Imbalance	External Validation	Confusion Matrix	Code Availability	Explainability
[16]	Low	High	Low	Low	High	High
[17]	Low	High	High	Low	High	Low
[18]	Low	High	Low	High	High	Low
[19]	Low	High	High	High	High	High
[20]	Low	Low	High	High	High	Low
[21]	Low	High	High	High	High	Low
[22]	Low	Low	Low	High	Low	Low
[23]	Low	High	Low	Low	High	High
[24]	Low	High	Low	High	High	Low
[25]	Low	Low	High	High	High	Low
[26]	Low	High	High	Low	Low	Low
[27]	Low	Low	Low	Low	High	Low
[28]	Low	Low	Low	High	High	Low
[29]	Moderate	High	Low	Low	High	High
[30]	Low	High	Moderate	High	Low	Low
[31]	Low	Low	High	High	Low	Low
[32]	Low	Low	High	Low	High	Low
[33]	Low	High	Moderate	High	Low	Low
[2]	Low	Low	Moderate	High	Low	Low
[34]	Low	High	Moderate	High	Low	High
[35]	Low	Low	Low	High	Low	Low
[11]	Low	Low	Moderate	High	Low	Low
[36]	Low	Low	Moderate	Low	Low	High
[37]	Low	High	Moderate	Low	Low	Low
[38]	Low	High	Moderate	High	Low	Low

## Data Availability

No new data were created or analyzed in this study. Data sharing is not applicable to this article.

## Supplementary Materials

The following supporting information can be downloaded at: https://www.mdpi.com/article/10.3390/toxics13121087/s1, Table S1: Preferred Reporting Items for Systematic Reviews and Meta-Analyses Extension for Scoping Reviews (PRISMA-ScR) Checklist. Ref. [57] is cited in Supplementary Materials.

## Abbreviations

The following abbreviations are used in this manuscript:

ML	Machine learning
hERG	human Ether-à-go-go-Related Gene
TdP	Torsades de Pointes
QSAR	Quantitative Structure-Activity Relationship
SVM	support vector machine
AUC-ROC	area under the receiver operating characteristic curve
TP	true positive
FP	false positive
TN	true negative
FN	false negative
XAI	explainable artificial intelligence
MCC	Matthews correlation coefficient
SIDER	Side Effect Resource
OMOP	Observational Medical Outcomes Partnership
LSER	linear solvation energy relationship
MOE	molecular operating environment
MACCS	Molecular Access System
ECFP	Extended Connectivity Fingerprints
RF	random forest
AGN	attention-based graph neural network
XGBoost	extreme gradient boosting
DICTrank	Drug-Induced Cardiotoxicity Rank
DIQTA	Drug-Induced QT Prolongation Atlas
EHRs	electronic health records

## References

[1] Ferri N., Siegl P., Corsini A., Herrmann J., Lerman A., Benghozi R. (2013). Drug attrition during pre-clinical and clinical development: Understanding and managing drug-induced cardiotoxicity. Pharmacol. Ther..

[2] Seal S., Spjuth O., Hosseini-Gerami L., Garcia-Ortegon M., Singh S., Bender A., Carpenter A.E. (2024). Insights into Drug Cardiotoxicity from Biological and Chemical Data: The First Public Classifiers for FDA Drug-Induced Cardiotoxicity Rank. J. Chem. Inf. Model..

[3] Onakpoya I.J., Heneghan C.J., Aronson J.K. (2016). Post-marketing withdrawal of 462 medicinal products because of adverse drug reactions: A systematic review of the world literature. BMC Med..

[4] Mamoshina P., Rodriguez B., Bueno-Orovio A. (2021). Toward a broader view of mechanisms of drug cardiotoxicity. Cell Rep. Med..

[5] Yang S., Kar S. (2023). Application of artificial intelligence and machine learning in early detection of adverse drug reactions (ADRs) and drug-induced toxicity. Artif. Intell. Chem..

[6] Zhang L., Tan J., Han D., Zhu H. (2017). From machine learning to deep learning: Progress in machine intelligence for rational drug discovery. Drug Discov. Today.

[7] Jo S.H., Hong H.K., Jung S.J., Chong S.H., Yun J.H., Koh Y.S., Choe H. (2007). Maprotiline block of the human ether-a-go-go-related gene (HERG) K+ channel. Arch. Pharm. Res..

[8] Doddareddy M.R., Klaasse E.C., Shagufta, Ijzerman A.P., Bender A. (2010). Prospective validation of a comprehensive in silico hERG model and its applications to commercial compound and drug databases. ChemMedChem.

[9] Ogura K., Sato T., Yuki H., Honma T. (2019). Support Vector Machine model for hERG inhibitory activities based on the integrated hERG database using descriptor selection by NSGA-II. Sci. Rep..

[10] Tang X., Wang Z., Hu S., Zhou B. (2022). Assessing Drug-Induced Mitochondrial Toxicity in Cardiomyocytes: Implications for Preclinical Cardiac Safety Evaluation. Pharmaceutics.

[11] Li S., Xu H., Liu F., Ni R., Shi Y., Li X. (2025). In silico prediction of drug-induced cardiotoxicity with ensemble machine learning and structural pattern recognition. Mol. Divers..

[12] Tosca E.M., Bartolucci R., Magni P., Poggesi I. (2021). Modeling approaches for reducing safety-related attrition in drug discovery and development: A review on myelotoxicity, immunotoxicity, cardiovascular toxicity, and liver toxicity. Expert Opin. Drug Discov..

[13] Khairnar S.I., Kulkarni Y.A., Singh K. (2022). Cardiotoxicity linked to anticancer agents and cardioprotective strategy. Arch. Pharm. Res..

[14] Grafton F., Ho J., Ranjbarvaziri S., Farshidfar F., Budan A., Steltzer S., Maddah M., Loewke K.E., Green K., Patel S. (2021). Deep learning detects cardiotoxicity in a high-content screen with induced pluripotent stem cell-derived cardiomyocytes. eLife.

[15] van Hasselt J.G.C., Rahman R., Hansen J., Stern A., Shim J.V., Xiong Y., Pickard A., Jayaraman G., Hu B., Mahajan M. (2020). Transcriptomic profiling of human cardiac cells predicts protein kinase inhibitor-associated cardiotoxicity. Nat. Commun..

[16] Yap C.W., Cai C.Z., Xue Y., Chen Y.Z. (2004). Prediction of torsade-causing potential of drugs by support vector machine approach. Toxicol. Sci..

[17] Xue Y., Li Z.R., Yap C.W., Sun L.Z., Chen X., Chen Y.Z. (2004). Effect of molecular descriptor feature selection in support vector machine classification of pharmacokinetic and toxicological properties of chemical agents. J. Chem. Inf. Comput. Sci..

[18] Bhavani S., Nagargadde A., Thawani A., Sridhar V., Chandra N. (2006). Substructure-based support vector machine classifiers for prediction of adverse effects in diverse classes of drugs. J. Chem. Inf. Model..

[19] Yang S.Y., Huang Q., Li L.L., Ma C.Y., Zhang H., Bai R., Teng Q.Z., Xiang M.L., Wei Y.Q. (2009). An integrated scheme for feature selection and parameter setting in the support vector machine modeling and its application to the prediction of pharmacokinetic properties of drugs. Artif. Intell. Med..

[20] Huang L.C., Wu X., Chen J.Y. (2011). Predicting adverse side effects of drugs. BMC Genom..

[21] He Y., Lim S.W., Yap C.W. (2012). Determination of torsade-causing potential of drug candidates using one-class classification and ensemble modelling approaches. Curr. Drug Saf..

[22] Wang Z., Clark N.R., Ma’ayan A. (2016). Drug-induced adverse events prediction with the LINCS L1000 data. Bioinformatics.

[23] Cai C., Fang J., Guo P., Wang Q., Hong H., Moslehi J., Cheng F. (2018). In Silico Pharmacoepidemiologic Evaluation of Drug-Induced Cardiovascular Complications Using Combined Classifiers. J. Chem. Inf. Model..

[24] Dey S., Luo H., Fokoue A., Hu J., Zhang P. (2018). Predicting adverse drug reactions through interpretable deep learning framework. BMC Bioinform..

[25] Amano Y., Honda H., Sawada R., Nukada Y., Yamane M., Ikeda N., Morita O., Yamanishi Y. (2020). In silico systems for predicting chemical-induced side effects using known and potential chemical protein interactions, enabling mechanism estimation. J. Toxicol. Sci..

[26] Llopis-Lorente J., Gomis-Tena J., Cano J., Romero L., Saiz J., Trenor B. (2020). In Silico Classifiers for the Assessment of Drug Proarrhythmicity. J. Chem. Inf. Model..

[27] Mamoshina P., Bueno-Orovio A., Rodriguez B. (2020). Dual Transcriptomic and Molecular Machine Learning Predicts all Major Clinical Forms of Drug Cardiotoxicity. Front. Pharmacol..

[28] Joshi P., Vedhanayagam M., Ramesh R. (2021). An Ensembled SVM Based Approach for Predicting Adverse Drug Reactions. Curr. Bioinform..

[29] Kadioglu O., Klauck S.M., Fleischer E., Shan L., Efferth T. (2021). Selection of safe artemisinin derivatives using a machine learning-based cardiotoxicity platform and in vitro and in vivo validation. Arch. Toxicol..

[30] Iftkhar S., de Sa A.G.C., Velloso J.P.L., Aljarf R., Pires D.E.V., Ascher D.B. (2022). cardioToxCSM: A Web Server for Predicting Cardiotoxicity of Small Molecules. J. Chem. Inf. Model..

[31] Ye L., Ngan D.K., Xu T., Liu Z., Zhao J., Sakamuru S., Zhang L., Zhao T., Xia M., Simeonov A. (2022). Prediction of drug-induced liver injury and cardiotoxicity using chemical structure and in vitro assay data. Toxicol. Appl. Pharmacol..

[32] Çelik F.K., Doğan S., Karaduman G. (2024). Drug-induced torsadogenicity prediction model: An explainable machine learning-driven quantitative structure-toxicity relationship approach. Comput. Biol. Med..

[33] Lin J., He Y., Ru C., Long W., Li M., Wen Z. (2024). Advancing Adverse Drug Reaction Prediction with Deep Chemical Language Model for Drug Safety Evaluation. Int. J. Mol. Sci..

[34] Vinh T., Nguyen L., Trinh Q.H., Nguyen-Vo T.H., Nguyen B.P. (2024). Predicting Cardiotoxicity of Molecules Using Attention-Based Graph Neural Networks. J. Chem. Inf. Model..

[35] He Y., Lv X., Long W., Zhai S., Li M., Wen Z. (2025). ToxBERT: An explainable AI framework for enhancing prediction of adverse drug reactions and structural insights. J. Pharm. Anal..

[36] Li T., Qu Y., Chen A., Thakkar S., Li D., Tong W. (2025). Beyond QSARs: Quantitative Knowledge-Activity Relationships (QKARs) for Enhanced Drug Toxicity Prediction. Toxicol. Sci..

[37] Qu Y., Li T., Liu Z., Tong W., Li D. (2025). DICTrank Is a Reliable Dataset for Cardiotoxicity Prediction Using Machine Learning Methods. Chem. Res. Toxicol..

[38] Rouen K.C., Narang K., Han Y., Wang D., Jang E., Brunkow S., Yarov-Yarovoy V., MacKerell A.D., Vorobyov I. (2025). Prediction of TdP Arrhythmia Risk Through Molecular Simulations of Conformation-specific Drug Interactions with the hERG K+, Na_V_1.5, and Ca_V_1.2 Channels. bioRxiv.

[39] De Ponti F., Poluzzi E., Montanaro N. (2001). Organising evidence on QT prolongation and occurrence of Torsades de Pointes with non-antiarrhythmic drugs: A call for consensus. Eur. J. Clin. Pharmacol..

[40] Karim A., Lee M., Balle T., Sattar A. (2021). CardioTox net: A robust predictor for hERG channel blockade based on deep learning meta-feature ensembles. J. Cheminform..

[41] Munawar S., Windley M.J., Tse E.G., Todd M.H., Hill A.P., Vandenberg J.I., Jabeen I. (2018). Experimentally Validated Pharmacoinformatics Approach to Predict hERG Inhibition Potential of New Chemical Entities. Front. Pharmacol..

[42] Kuhn M., Letunic I., Jensen L.J., Bork P. (2016). The SIDER database of drugs and side effects. Nucleic Acids Res..

[43] Ryan P.B., Schuemie M.J., Welebob E., Duke J., Valentine S., Hartzema A.G. (2013). Defining a reference set to support methodological research in drug safety. Drug Saf..

[44] Qu Y., Li T., Liu Z., Li D., Tong W. (2023). DICTrank: The largest reference list of 1318 human drugs ranked by risk of drug-induced cardiotoxicity using FDA labeling. Drug Discov. Today.

[45] Klon A.E. (2010). Machine learning algorithms for the prediction of hERG and CYP450 binding in drug development. Expert Opin. Drug Metab. Toxicol..

[46] Li S., Xu Z., Guo M., Li M., Wen Z. (2022). Drug-induced QT Prolongation Atlas (DIQTA) for enhancing cardiotoxicity management. Drug Discov. Today.

[47] Guo W., Liu J., Dong F., Song M., Li Z., Khan M.K.H., Patterson T., Hong H. (2023). Review of machine learning and deep learning models for toxicity prediction. Exp. Biol. Med..

[48] Falcón-Cano G., Morales-Helguera A., Lambert H., Cabrera-Pérez M., Molina C. (2025). hERG toxicity prediction in early drug discovery using extreme gradient boosting and isometric stratified ensemble mapping. Sci. Rep..

[49] Hossain D., Al Abir F., Chen J.Y. (2025). hERG-LTN: A New Paradigm in hERG Cardiotoxicity Assessment Using Neuro-Symbolic and Generative AI Embedding (MegaMolBART, Llama3.2, Gemini, DeepSeek) Approach. bioRxiv.

[50] Jing Y., Zhang Y., Zhao G., McGuire T., Zhao J., Gibbs B., Hou G., Feng Z., Xue Y., Xie X.Q. (2025). GraphDeep-hERG: Graph Neural Network PharmacoAnalytics for Assessing hERG-Related Cardiotoxicity. Pharm. Res..

[51] Mayr A., Klambauer G., Unterthiner T., Hochreiter S. (2016). DeepTox: Toxicity prediction using deep learning. Front. Environ. Sci..

[52] Cavasotto C.N., Scardino V. (2022). Machine Learning Toxicity Prediction: Latest Advances by Toxicity End Point. ACS Omega.

[53] van Berlo J.H., Maillet M., Molkentin J.D. (2013). Signaling effectors underlying pathologic growth and remodeling of the heart. J. Clin. Investig..

[54] Hu Q., Li J., Li X., Zou D., Xu T., He Z. (2024). Machine learning to predict adverse drug events based on electronic health records: A systematic review and meta-analysis. J. Int. Med. Res..

[55] International Conference on Harmonisation (2005). The Non-Clinical Evaluation of the Potential for Delayed Ventricular Repolarization (QT Interval Prolongation) by Human Pharmaceuticals. https://www.ich.org/page/safety-guidelines.

[56] U.S. Food and Drug Administration (2025). Artificial Intelligence-Enabled Device Software Functions: Lifecycle Management and Marketing Submission Recommendations. https://www.fda.gov/regulatory-information/search-fda-guidance-documents/artificial-intelligence-enabled-device-software-functions-lifecycle-management-and-marketing.

[57] Tricco A.C., Lillie E., Zarin W., O’Brien K.K., Colquhoun H., Levac D., Moher D., Peters M.D.J., Horsley T., Weeks L. (2018). PRISMA Extension for Scoping Reviews (PRISMAScR): Checklist and Explanation. Ann. Intern. Med..

